# Impact of posthemorrhagic ventricular dilatation on cerebral oxygenation in preterm infants with intraventricular hemorrhage

**DOI:** 10.1038/s41390-025-04738-y

**Published:** 2026-01-08

**Authors:** Julia Elis, Lisa Klein, Mirjam Steiner, Katharina Moser, Vito Giordano, Gabriel A. Vignolle, Lucia Ciglar, Gregor Kasprian, Georg Langs, Monika Olischar, Angelika Berger, Katharina Goeral

**Affiliations:** 1 Department of Pediatrics and Adolescent Medicine, Division of Neonatology, Intensive Care and Neuropediatrics, Full Member of ERN EpiCare, Medical University of Vienna - Comprehensive Center for Pediatrics, Vienna, Austria; 2 Center for Health & Bioresources, Competence Unit Molecular Diagnostics, AIT Austrian Institute of Technology GmbH, Vienna, Austria; 3 Department of Biomedical Imaging and Image-guided Therapy, Division of Neuroradiology and Musculoskeletal Radiology, Medical University of Vienna, Vienna, Austria; 4 Department of Biomedical Imaging and Image-guided Therapy, Computational Imaging Research Lab, Medical University of Vienna, Vienna, Austria

## Abstract

**Background:**

To assess the longitudinal effects of intraventricular hemorrhage (IVH) and posthemorrhagic ventricular dilatation (PHVD) on cerebral oxygenation using near-infrared spectroscopy (NIRS).

**Methods:**

This prospective cohort study included preterm neonates born <34 weeks’ gestation between 2013 and 2024. Regional cerebral oxygen saturation (rScO_2_) was measured from IVH diagnosis until term-equivalent age. Duration of abnormal rScO_2_ values (<55%; >85%) and cerebral fractional tissue oxygen extraction (cFTOE) were analyzed.

**Results:**

A total of 154 preterm infants with IVH (median gestational age: 25^+4^ weeks) were included, of whom 65 (42.2%) developed PHVD, with 56 (86.2%) requiring temporizing neurosurgical intervention. Analysis of over 30,000 hours of NIRS data revealed a significant decline in cerebral oxygenation with increasing IVH severity (*p* = 0.023). Infants with PHVD had lower rScO₂ (*p* < 0.001), spent more time with rScO_2_ < 55% (*p* < 0.001), and exhibited higher cFTOE (*p* < 0.001) than those without PHVD. Within the PHVD group, more interventions were associated with lower rScO_2_ levels (*p* = 0.010) and higher cFTOE values (*p* = 0.005).

**Conclusion:**

IVH and PHVD profoundly impair cerebral oxygenation. High-grade IVH leads to rScO_2_ deterioration, further exacerbated in infants with greater PHVD burden. These findings highlight the need for targeted strategies to stabilize cerebral oxygenation in this vulnerable population.

**Impact:**

- Cerebral oxygenation declines with increasing intraventricular hemorrhage (IVH) severity in preterm infants.
- Posthemorrhagic ventricular dilatation (PHVD) severity, reflected by the number of neurosurgical interventions, is linked to worsening cerebral oxygenation and increased oxygen extraction.
- This prospective cohort study provides a comprehensive longitudinal dataset using near-infrared spectroscopy (NIRS) to link IVH severity and PHVD to cerebral oxygenation dynamics.
- Findings underscore the urgent need for targeted neuroprotective interventions to stabilize brain oxygenation in preterm infants with severe IVH and PHVD.

## Introduction

Despite advances in perinatal and neonatal care, preterm birth remains a major global health concern, associated with high morbidity and mortality rates.^1^ Among preterm neonates, intraventricular hemorrhage (IVH) is one of the most common brain injuries, occurring in 25–30%.^2,3^ IVH originates in the subependymal germinal matrix and is most commonly classified using the ultrasound-based Papile system, which categorizes into grades I to IV depending on location and severity.^4–6^ However, grade IV was later redefined by Volpe as periventricular hemorrhagic infarction (PVHI), a distinct parenchymal venous infarction that may occur alongside any ipsilateral IVH grade.^7^

A major complication of IVH is the progressive development of posthemorrhagic ventricular dilatation (PHVD), which affects ~50% of infants with grade III or PVHI.^8^ PHVD may lead to elevated intracranial pressure, inflammation-induced free radical damage, and white matter injury, contributing to adverse neurodevelopmental outcome, including cerebral palsy, cognitive impairment and developmental delays that persist into childhood and beyond.^9,10^

To manage PHVD, various interventions, including lumbar punctures, temporizing neurosurgical interventions (tNSIs) like external ventricular drains (EVD) or ventricular reservoirs (e.g., Ommaya reservoirs), and permanent ventriculoperitoneal shunts, are employed to decompress the ventricles.^8,10^ However, the optimal timing and type of intervention remain debated, as clinicians must balance surgical risks with the potential harm of increasing ventricular size.^11^

Near-infrared spectroscopy (NIRS), a non-invasive tool that measures cerebral oxygenation based on the differential absorption of near-infrared light by oxyhemoglobin and deoxyhemoglobin, has gained clinical recognition as it offers unique insights into functional cerebrovascular consequences that complement structural imaging.^12–14^ Studies investigating cerebral oxygenation dynamics over short timeframes have shown that IVH alters cerebral autoregulation, leading to a discrepancy between oxygen demand and delivery.^15,16^ One study reported prolonged decreases in regional cerebral oxygen saturation (rScO_2_) and increased cerebral fractional tissue oxygen extraction (cFTOE) within the first 70 days in infants with IVH compared to those without.^17^ In addition to the impact of IVH, several studies have demonstrated short-term changes in infants with PHVD, with a transient improvement in rScO_2_ after ventricular decompression.^13,14^

Nevertheless, the longitudinal influence of IVH and PHVD on cerebral oxygenation in these patients remains insufficiently studied and poorly understood. This study investigates how cerebral oxygenation correlates with IVH severity and progressive PHVD, using the number of tNSIs required as an indirect marker of PHVD burden.

## Methods

### Patients

This prospective cohort study included preterm neonates with IVH born <34 weeks of gestational age (GA) at the Medical University of Vienna between July 2013 and April 2024. Written informed consent was obtained from all parents/legal guardians. Neonates with chromosomal anomalies, congenital or central nervous system malformations were excluded.

### Clinical practices

PHVD was managed stepwise according to El-Dib et al.^10^. Until 2020, lumbar punctures were not routinely used in our cohort. From 2020 onward, they were systematically introduced as a first-line temporizing measure once the ventricular measurements met the following criteria: ventricular index exceeded the 97th percentile and either anterior horn width >6 mm or thalamo-occipital distance >25 mm, plus evidence of a documented upward trajectory, consistent with the mentioned risk stratification tool. Lumbar punctures were repeated until stabilization or escalation was required.

tNSIs were performed when ventricular dilatation progressed despite lumbar punctures (ventricular index >97th percentile plus 4 mm) or when persistent clinical symptoms were present. These interventions allowed ongoing cerebrospinal fluid (CSF) drainage until stabilization or the need for permanent treatment. For this study, tNSIs were defined strictly at the procedure level, i.e., the placement of an EVD or Ommaya reservoir, regardless of drainage days or number of taps. Infants weighting <1000 g typically received an EVD, whereas infants ≥1000 g usually received an Ommaya reservoir, with occasional exceptions due to the timing of the intervention and availability of neurosurgical expertise.

Permanent treatment consisted exclusively of ventriculoperitoneal shunt placement, which was performed if ventricles re-expanded post-removal of tNSI or if persistent high CSF flow rates were observed. Reservoir taps were stopped a few days before the scheduled shunt placement; a slight increase in ventricular size during this interval is expected and facilitates safe shunt implantation, and no clinical difficulties occurred. Institutional criteria for ventriculoperitoneal shunt placement included ongoing ventricular enlargement, clinical signs of raised intracranial pressure or neurological compromise, a minimum body weight of 2000 g, and CSF protein levels <200 mg/dL. Importantly, all infants received tNSIs before shunt placement; no infant proceeded directly to permanent shunting. Endoscopic third ventriculostomy was not part of our institutional practice and was not performed in this cohort.

All neurosurgical procedures were performed by specialized neurosurgeons under general anesthesia, typically requiring intubation and ventilation.

### Near-infrared spectroscopy

NIRS monitoring (INVOS^TM^ 5100C Cerebral/Somatic Oximeter Monitor; Covidien) with a neonatal sensor placed frontoparietally started at the time of IVH diagnosis (sampling rate: 0.2 Hz). Neonates with IVH were monitored twice weekly for ≥4 h until 32 weeks corrected age, then every 2 weeks until term-equivalent age. In infants with progressive PHVD, monitoring was near-continuous once they reached the yellow or red line as defined by El-Dib et al.^10^ until they either returned to the green line or surgical intervention occurred. To enable comparison with previously published studies that used adult sensors (SAFB-SM) in neonatal populations, neonatal rScO_2_ values were converted using: rScO_2_adult = (rScO_2_neonate–19.11)/0.8481.^18^ We did not, however, perform direct comparisons with adult sensor data in this study.

We assessed time outside the established normal rScO_2_ range (<55% or >85%)^19^ and evaluated periods of substantial fluctuations in cerebral oxygenation, an indicator for impaired autoregulation, defined as deviations exceeding 10% from the hourly rScO_2_ baseline.^20^ Additionally, continuously recorded values of pulsoximetric arterial oxygen saturation (SaO_2_) were employed to calculate cFTOE for each data point: cFTOE = (SaO_2_-[rScO_2_])/SaO_2_, with a normative range of 0.17–0.42.^18,21,22^ We also examined the relationship between mean arterial blood pressure (MAP) and rScO₂ to explore autoregulatory capacity, using correlation and mixed-effects models across different time windows.

### Data cleaning and processing

To clean the data and remove potential artifacts, an algorithm was applied using Matlab (ver. R2019b, Mathworks, Inc., Natick, MA). In the initial phase, missing values were detected, and values that fell below the detection threshold of the device (rScO_2_ < 15%) were excluded from analysis. In a second step, an overlapping 1-h sliding window was used to exclude data points with a greater than 2.5 standard deviation from the mean rScO_2_ value in each window, following previously established methods.^23^

Finally, after the previous cleaning steps, data points were excluded if 90% or more of the values within a 1-h window were missing. Missingness was most commonly due to motion artefacts, electrical disturbances, or unstable sensor attachment, but could also occur when a monitoring session was terminated early within a given hour. This ensured that at least 10% of data points were available within each 1-h window for analysis. All rejected or missing data points were replaced with not-a-number (NaN) and not part of statistical analysis.

### Data analysis

Due to the complexity of NIRS parameter trajectories, which are not adequately represented by traditional linear or non-linear regression models, this study employed a conditional mean function for visualization (ggplot2 package within the R statistical package) (R Foundation for Statistical Computing, Vienna, Austria).^17,24^ Given the features of the dataset, in which cleaned raw second-to-second data were averaged into daily means per patient, the number of daily means per analyzed subgroup comprised less than 1000 data points. To model the relationships between NIRS parameters and postnatal age in these subgroups, a locally estimated scatterplot smoothing (LOESS) approach was employed. For graphical representation, error-corrected parameters were plotted against age, with the line of best fit and the 95% confidence intervals.

### Statistical analysis

To gain more insights into the correlation of NIRS parameters and the severity of IVH, patients were initially grouped according to their IVH grade. In general, low-grade IVH refers to grades I and II, whereas high-grade IVH includes grade III and PVHI. For this study, all PVHI cases were classified together as high-grade IVH regardless of the accompanying IVH grade, as our focus was on the presence of parenchymal venous infarction. Subsequently, differences between patients with IVH and patients who developed progressive PHVD (IVH+PHVD) were investigated. To assess the impact of progressive PHVD on cerebral oxygenation, these patients were further categorized based on PHVD burden, represented by the number of tNSI.

Categorical variables were compared using Chi-square test; NIRS parameters using Mann–Whitney U or Kruskal–Wallis test. Analysis was performed using SPSS version 23 (IBM Corporation) with a two-sided *p*-value of <0.05 considered statistically significant.

### Statement of ethics

This study was approved by the local ethics committee (Ethics Committee of the Medical University of Vienna) (EK 252/2011 and EK 1677/2022). All procedures were conducted in accordance with the Declaration of Helsinki.

## Results

### Demographics and clinical characteristics

During the 11-year study period, 376 neonates born <34 weeks of gestation with IVH were treated at the Medical University of Vienna. After excluding 26 due to predefined criteria, 350 were considered eligible, with IVH grades distributed as follows: grade I: 74 (21.1%), II: 94 (26.9%), III: 102 (29.1%) and PVHI: 80 (22.9%).

Of these, 142 had no NIRS data due to technical issues. Additionally, 14 neonates died before day 2, 19 were transferred from other hospitals more than 7 days after birth, leaving no early NIRS data accessible, and 21 lacked sufficient measurements, leaving 154 neonates included in the final analysis.

Median GA was 25^+4^ (24^+1^–27^+1^) weeks, birthweight 805 (630–975) g (Table 1). IVH was diagnosed at a median of 2 days (1–3), with a median IVH grade of III (II-PVHI) and the severity distributed as follows: 12 patients (7.8%) presented grade I, 28 (18.2%) II, 65 (42.2%) III and 49 (31.8%) PVHI. Among all patients, 89 (57.8%) did not develop PHVD, whereas 65 (42.2%) progressed to PHVD (17.9% grade II, 55.4% grade III, 49.0% PVHI affected). Of these 65 patients, 23 (35,4%) received at least one lumbar puncture. Among them, 9 infants (39.1%, corresponding to 13.8% of all PHVD cases) were managed exclusively with lumbar punctures (median of 2 (1–7); range 1–8 procedures), as their ventricular dilatation remained subtle and did not progress to the high-risk (red) line.^10^ Their ventricular size stabilized without the need for device-based intervention, corresponding to cases often described as apparent spontaneous arrest of PHVD. In contrast, 56 (86.2%) ultimately required tNSI, such as EVD or Ommaya. Within this neurosurgical intervention group, 24 (42.8%) underwent a single tNSI, whereas 32 (57.1%) required multiple tNSIs, with a median of 3 (3–4) procedures, independent of drainage duration or number of taps. The need for multiple tNSIs was most commonly due to catheter malposition, catheter migration, obstruction by blood or debris, or concerns for infection. The first tNSI was performed at day 17 (14–21), the second at day 45 (34–56). Temporizing measures were maintained for a median of 20.5 days (15.0–29.3). Four patients died shortly after device insertion. Following tNSI, 29 patients (51.8%) required a ventriculoperitoneal shunt, which was performed at a median of 85 days (67–101), corresponding to a median postnatal age of 37^+3^ (36^+0^–39^+4^; range 34^+0^–41^+5^). At shunt placement, the median body weight was 2.62 kg (2.24–3.02; range 2.00–3.22 kg).

**Table 1:** Clinical description of the study cohort.

	All patients (*n* = 154)	IVH* (*n* = 89)	IVH + PHVD** (*n* = 65)	*p*
Male, *n* (%)	100 (64.9)	57 (64.0)	43 (66.2)	0.79
Inborn, *n* (%)	132 (85.7)	83 (93.3)	49 (75.4)	**0.002**
Birth mode, *n* (%)				
Spontaneous delivery	36 (23.4)	23 (25.8)	13 (20.0)	0.56
C-section	93 (60.4)	53 (59.6)	40 (61.5)	
Emergency C-section	24 (15.6)	13 (14.6)	11 (16.9)	
Home delivery	1 (0.6)	0 (0.0)	1 (1.5)	
GA, weeks^+days^, median (IQR)	25^+4^ (24^+1^–27^+1^)	25^+0^ (23^+5^–26^+4^)	26^+3^ (24^+6^–28^+3^)	**<0.001**
22^+6 ^– 24^+6^ weeks, *n* (%)	60 (39.0)	44 (49.4)	16 (24.6)	
25^+0^ – 27^+6^ weeks, *n* (%)	64 (41.6)	36 (40.4)	28 (43.1)	
28^+0 ^– 30^+6^ weeks, *n* (%)	22 (14.3)	7 (7.9)	15 (23.1)	
31^+0^ – 33^+6^ weeks, *n* (%)	8 (5.2)	2 (2.2)	6 (9.2)	
Multiple birth, *n* (%)	42 (26.6)	23 (25.8)	18 (27.7)	0.43
Measurements at birth				
Weight (g), median (IQR)	805 (630–975)	710 (585–885)	870 (760–1213)	**<0.001**
Weight percentile, median (IQR)	53 (28–74)	50 (27–72)	60 (31–82)	0.30
Length percentile, median (IQR)	56 (36–75)	53 (32–73)	59 (41–80)	0.19
Head circumference percentile*, median (IQR)	60 (32–81)	58 (29–78)	61 (41–87)	0.20
APGAR score				
1 min, median (IQR)	7 (5–8)	7 (5–8)	7 (4–8)	0.28
5 min, median (IQR)	8 (7–9)	8 (7–9)	8 (7–9)	0.45
10 min, median (IQR)	9 (8–9)	9 (8–9)	9 (8–9)	0.24
Umbilical cord pH, median (IQR)	7.30 (7.23-–7.36)	7.31 (7.24–7.37)	7.29 (7.19–7.36)	0.29
Neonatal diagnoses				
Patent Ductus Arteriosus with surgical closure, *n* (%)	12 (7.8)	7 (7.9)	5 (7.7)	0.68
Bronchopulmonary dysplasia < 36 weeks GA, *n* (%)	10 (6.5)	7 (7.9)	3 (4.6)	0.33
Necrotizing enterocolitis with surgical treatment, *n* (%)	11 (7.1)	7 (7.9)	4 (6.2)	0.91
IVH grade, median (IQR)	III (II-PVHI)	III (II-PVHI)	III (III-PVHI)	**<0.001**
Grade I, *n* (%)	12 (7.8)	12 (13.5)	0 (0.0)	
Grade II, *n* (%)	28 (18.2)	23 (25.8)	5 (7.7)	
Grade III, *n* (%)	65 (42.2)	29 (32.6)	36 (55.4)	
PVHI, *n* (%)	49 (31.8)	25 (28.1)	24 (36.9)	
Age (days) at IVH diagnosis, median (IQR)	2.0 (1.0–3.0)	2.0 (1.0–4.0)	2.0 (1.0–3.0)	0.59
Clinical course				**0.017**
Survival until term, *n* (%)	118 (76.6)	62 (69.7)	56 (86.2)	
Death, *n* (%)	36 (23.4)	27 (30.3)	9 (13.8)	

Bold *p*-values indicate statistically significant group differences (*p*<0.05).

*IVH includes all grades of intraventricular hemorrhage.

**IVH+PHVD refers to progressive ventricular dilatation.

### NIRS analysis

A total of 2211 NIRS measurements from 154 patients were available for analysis, with a mean recording length of 13.4 (±4.7) hours, totaling ~29,707 h. IVH-only patients underwent a mean of 90 h/patient, PHVD patients 334 h/patient, reflecting our structured monitoring protocol, which mandates more frequent and prolonged recordings in infants with PHVD once they exceed the green line thresholds as defined by El-Dib et al.^10^ Importantly, despite different recording durations, key timepoints—including both event-based (e.g., day of IVH diagnosis and subsequent days, and several PHVD-related windows (before/at tNSI, early after tNSI, up to 40 days after a tNSI, and >40 days after tNSI) in IVH+PHVD patients) and age-based timeframes (e.g., 28 days, 32 weeks gestational age, term-equivalent age)— were systematically captured across groups, allowing for meaningful comparisons.

### Cerebral oxygenation in IVH and PHVD (Table 2)

rScO_2_ levels declined with increasing IVH grade (*p* = 0.023): patients with IVH grade I had a median of 74.2%, decreasing by 7.5% in grades II/III and 12.2% in PVHI. The percentage of time with rScO_2_ < 55% increased with IVH severity (*p* = 0.009), and was highest in PVHI, where infants spent a median of 9.9% of time under this threshold. cFTOE did not differ across groups.

**Table 2:** Comparison of NIRS parameters, presented as median (IQR) between patients with different IVH grades and IVH patients with and without PHVD.

		IVH	IVH Grade I (*n* = 12)	IVH Grade II (*n* = 23)	IVH Grade III (*n* = 29)	PVHI (*n* = 25)	p*	high-grade IVH+PHVD	IVH Grade III +PHVD (*n* = 36)	PVHI +PHVD (*n* = 24)	p^**^
**rScO**_**2**_ **(%)** neonatal		66.37 (60.91–73.53)	74.24 (65.03–80.19)	67.55 (63.33–74.68)	65.85 (57.13–70.84)	62.07 (54.25–71.49)	**0.023**	56.03 (45.74–62.02)	57.85 (46.44–62.21)	51.72 (37.74–58.11)	**<0.001**
**rScO**_**2**_ **(%)** adult^a^		55.72 (49.28–64.17)	65.01 (54.14–72.02)	57.11 (52.19–65.53)	55.11 (45.84–60.99)	50.66 (41.94–61.76)	**0.024**	43.69 (31.78–50.60)	45.68 (32.34–50.82)	38.45 (25.89–45.99)	**<0.001**
**cFTOE** neonatal		0.29 (0.20–0.35)	0.20 (0.14–0.30)	0.27 (0.20–0.32)	0.29 (0.23–0.39)	0.32 (0.22–0.42)	0.14	0.40 (0.35–0.52)	0.39 (0.35–0.52)	0.46 (0.38–0.60)	**<0.001**
**cFTOE** adult		0.39 (0.30–0.47)	0.30 (0.23–0.42)	0.39 (0.32–0.46)	0.40 (0.33–0.53)	0.45 (0.33–0.55)	0.15	0.55 (0.48–0.67)	0.51 (0.47–0.67)	0.60 (0.51–0.72)	**<0.001**
**Time out of normal range (%)**	<**55%**	4.59 (0.05–16.46)	0.66 (0.00–4.91)	3.37 (0.03–11.25)	7.98 (0.21–34.62)	9.93 (1.21–41.47)	**0.009**	36.87 (11.51–85.36)	24.79 (5.18–81.95)	70.35 (22.80–92.46)	**<0.001**
	>**85%**	0.00 (0.00–1.76)	0.10 (0.00–15.50)	0.02 (0.00–1.26)	0.00 (0.00–2.67)	0.00 (0.00–0.90)	0.44	0.00 (0.00–0.02)	0.00 (0.00–0.02)	0.00 (0.00–0.01)	0.07
**Fluctuation (%)**		1.29 (0.38–3.14)	2.02 (0.08–4.16)	2.08 (0.52–3.30)	1.41 (0.51–2.76)	1.03 (0.39–2.08)	0.44	1.84 (0.91–3.76)	1.47 (0.71–3.26)	3.0 (1.11–5.47)	0.06

Bold *p*-values indicate statistically significant group differences (*p*<0.05).

*p-value for comparison of median values among patients with IVH grades I, II, III, PVHI.

**p-value for comparison of median values between patients with IVH grades III and PVHI, with and without PHVD.

^a^rScO_2_adult = (rScO_2_neonatal − 19.11)/0.8481^18^.

Among IVH grade III and PVHI, PHVD presence further reduced rScO_2_ by a total of 9.2% and increased time <55% rScO_2_ (both *p* < 0.001). For instance, in IVH with PVHI, infants without PHVD had a median of 9.9% of time with rScO₂ <55%, whereas infants with IVH+PHVD showed a median of 70.4%. A similar pattern was observed for cFTOE which increased by 12.0% (*p* < 0.001) with PHVD.

**Fig. 1:**
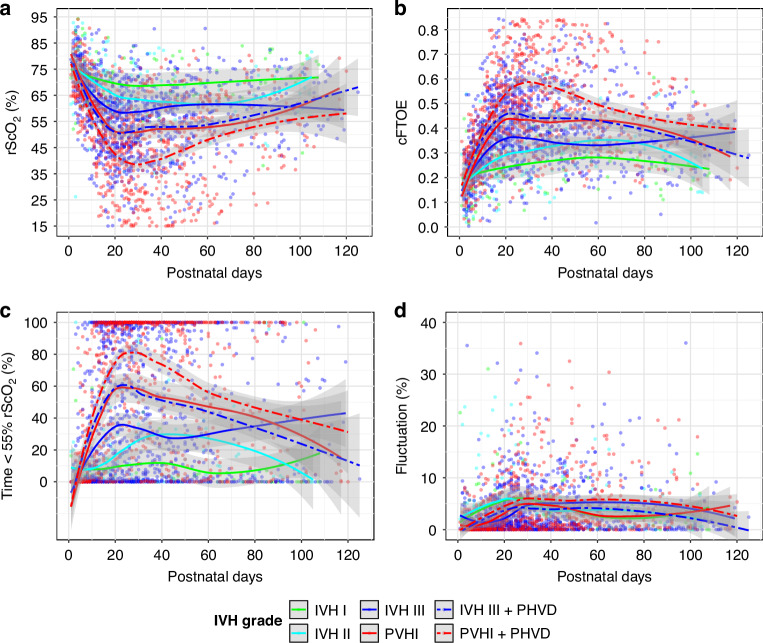
Trajectories of NIRS parameters by postnatal days and IVH severity. Trajectory of rScO_2_ (**a**), cFTOE (**b**), Time <55% rScO_2_ (**c**), and Fluctuation (**d**) plotted by postnatal age (in days) for patients with IVH: grade I, II, III, and PVHI. Solid/dashed lines indicate LOESS non-linear regression curves, with gray shading representing the 95% confidence interval.

When analyzing the combined effects of IVH severity and PHVD on cerebral oxygenation, IVH-only patients experienced a 3.8% decrease in rScO_2_ from grade III to PVHI, versus 6.1% with PHVD. Similarly, cFTOE rose by 0.03 vs. 0.07, and time of rScO_2_ < 55% increasing by 2.0% vs. 45.6%. Trajectories were visualized over postnatal age (Fig. 1a–d).

### Cerebral oxygenation in relation to PHVD burden

PHVD patients were categorized according to the number of tNSIs required to manage the progression of their ventricular dilatation (Table 3). rScO_2_ decreased with increasing numbers of tNSIs (*p* = 0.010): no tNSI: 63.5%, while it decreased by about 5.8% in patients with a single tNSI, and by about 9.7% in patients with multiple tNSIs. cFTOE values increased, indicating higher cerebral oxygen extraction (0.32 vs. 0.39 vs. 0.43, *p* = 0.005) with more tNSI.

**Table 3:** Comparison of NIRS parameters, presented as median (IQR), between neonates with IVH+PHVD and no tNSI, a single tNSI and multiple tNSIs.

		no tNSI^b^ (*n* = 9)	single tNSI (*n* = 24)	multiple tNSIs (*n* = 32)	*p*	p*	p**	p***
**rScO**_**2**_ **(%)** neonatal		63.54 (52.9–73.2)	57.75 (46.43–64.01)	53.81 (43.03–60.54)	**0.010**	0.116	0.163	**0.002**
**rScO**_**2**_ **(%)** adult^a^		52.38 (39.96–63.94)	45.56 (32.26–54.13)	41.03 (30.09–48.84)	**0.010**	0.113	0.165	**0.002**
**cFTOE** neonatal		0.32 (0.20–0.43)	0.39 (0.31–0.52)	0.43 (0.37–0.54)	**0.005**	0.063	0.202	**0.001**
**cFTOE** adult		0.46 (0.32–0.60)	0.51 (0.42–0.67)	0.57 (0.51–0.69)	**0.006**	0.067	0.199	**0.003**
**Time out of normal range (%)**	<**55%**	20.99 (1.19–52.97)	22.64 (4.04–85.23)	51.53 (19.08–88.3)	0.05	0.415	0.129	**0.019**
	>**85%**	0.00 (0.00–6.16)	0.00 (0.00–0.00)	0.0 (0.0–0.02)	0.10	0.056	0.741	0.07
**Fluctuation (%)**		1.91 (1.08–2.69)	1.10 (0.39–2.70)	3.06 (1.22–5.08)	**0.013**	0.185	**0.003**	0.15

Bold *p*-values indicate statistically significant group differences (*p*<0.05).

*p-value for comparison of median values between patients with no tNSI and single tNSI.

**p-value for comparison of median values between patients with single tNSI and multiple tNSIs.

***p-value for comparison of median values between patients with no tNSI and multiple tNSIs.

^a^rScO_2_adult = (rScO_2_neonatal − 19.11)/0.8481^18^.

^b^These infants with PHVD stabilized after lumbar punctures without requiring device-based intervention (apparent spontaneous arrest).

The time of rScO_2_ < 55% rose significantly (21.0% vs. 22.6% vs. 51.5%, *p* = 0.019) with more tNSI. Fluctuations were higher in the group with multiple tNSIs compared to the group with a single tNSI (3.1% vs. 1.1%, *p* = 0.003).

**Fig. 2:**
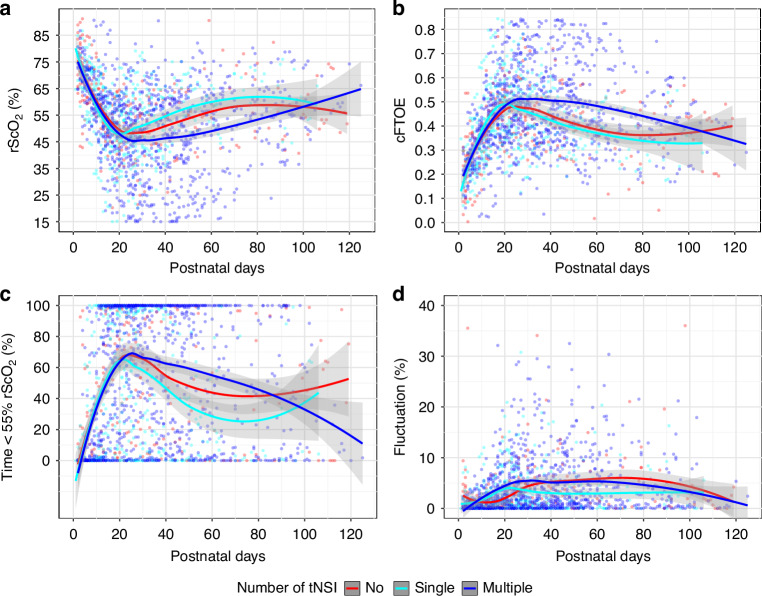
Trajectories of NIRS parameters by postnatal days and PHVD burden. Trajectory of rScO_2_ (**a**), cFTOE (**b**), Time <55% rScO_2_ (**c**), and Fluctuation (**d**) plotted by postnatal age (in days) for patients with IVH+PHVD and no temporizing neurosurgical intervention (tNSI), a single tNSI and multiple tNSIs. Solid/dashed lines indicate LOESS non-linear regression curves, with gray shading representing the 95% confidence interval.

No significant differences were found between no tNSI and single tNSI. As shown in Fig. 2a–d, the trajectories of rScO_2_ and cFTOE over time suggest that these two groups follow a similar trend.

### Association between blood pressure and cerebral oxygenation

Across the entire study period, we observed a significant inverse correlation between MAP and rScO_2_ (*p* < 0.001). This association was most evident during the acute IVH phase (first week after diagnosis; *p* < 0.001). In contrast, no significant correlation was detected during the acute PHVD phase (−2 to +8 days around the first tNSI; *p* = 0.916) or later follow-up (+39 days; *p* = 0.977). The corresponding trajectories of MAP and rScO_2_ across these time frames are shown in Supplementary Fig. S1A–D.

## Discussion

This study offers one of the most extensive longitudinal assessments of cerebral oxygenation in IVH and PHVD, spanning nearly 20 weeks. Using the number of tNSI as a proxy for PHVD burden, we found that both IVH severity and progressive PHVD significantly impair cerebral oxygenation. The distribution of PHVD across IVH grades in our cohort was consistent with the literature.^8,25^ A small subgroup of infants (about one in seven with PHVD) stabilized after lumbar punctures without requiring device-based intervention, corresponding to the cases often described as apparent spontaneous arrest of PHVD.

Early declines in rScO_2_ and elevated cFTOE values during the first 72 h of life are known,^18,26^ but long-term patterns remain underexplored. Our findings show a progressive decline in rScO_2_ with increasing IVH severity, with infants with PVHI spending significantly more time below the critical 55% threshold, indicating persistent cerebral hypoxemia and an elevated risk of secondary brain injury.^17^

These differences gradually diminished by week 10, aligning with earlier reports of desaturation lasting up to 70 days.^17,27^ In the study by Vesoulis et al.^17^, infants with IVH exhibited a biphasic course, with an initial decline, plateau, and partial recovery around day 62. Their supplementary analysis also suggested recovery in infants with PHVD, but the subgroup was small, and management details were not provided. In our cohort, we observed partial recovery beyond 40 days after tNSI, likely reflecting a combination of ventricular decompression through neurosurgical intervention and ongoing cerebral maturation. However, as this is an observational cohort study, causality cannot be established. Interestingly, no overall significant differences in cFTOE levels were observed between IVH grades. However, a closer examination of the weekly medians revealed substantial differences between weeks 3 and 6, with infants having higher IVH grades exhibiting increased extraction rates.

These findings support impaired cerebral autoregulation in IVH, consistent with the vulnerability of the preterm cerebrovascular system.^15,16,28^ The prolonged periods of low rScO₂ indicate sustained metabolic stress in vulnerable white matter regions,^17^ and the significant inverse correlation between MAP and rScO₂ during the acute IVH phase further reflects compromised autoregulatory capacity. In later stages, when PHVD emerges and interventions are performed, other factors such as ventricular size, intracranial pressure, or chronic metabolic alterations may dominate cerebral oxygenation dynamics. This is in line with our observation that between-patient variance explained most of the differences in the PHVD phases.

PHVD contributes to secondary brain injury through mechanical distortion and inflammation-driven toxicity from intraventricular blood.^9,29–32^ Compared to IVH only, patients with IVH+PHVD exhibited significantly worse NIRS parameters. We propose that this deterioration reflects two distinct but potentially synergistic mechanisms. First, circulatory disturbance appears to be an early and fundamental pathophysiological consequence of IVH, affecting all subgroups to varying degrees. Likely drivers include inflammation-mediated endothelial dysfunction and impaired autoregulation, consistent with our observation of an inverse correlation between MAP and rScO₂ during the acute IVH phase. These vulnerabilities may be aggravated by systemic factors such as anemia, arterial partial pressure of carbon dioxide, sepsis, or ductus arteriosus.^4,33,34^ Second, in patients with progressive PHVD, these vascular disturbances are compounded by mechanical effects of increased intracranial pressure, explaining the most profound alterations observed in NIRS parameters.^26,35,36^

Further analysis of PHVD, using the number of tNSIs as a surrogate marker, revealed a clear association between PHVD burden and cerebral oxygenation. This finding corroborates prior studies demonstrating an inverse correlation between cerebral oxygen saturation and ventricular size, although these studies were conducted with a smaller cohort and used only weekly NIRS measurements.^12,14^

Given the significant neurodevelopmental implications and long-lasting consequences of PHVD on the cerebral oxygenation shown in this study, it is crucial to determine the optimal timing and type of intervention to minimize the risk of secondary brain injury.

Treatment strategies for PHVD should prioritize the prevention of additional injury, a link that has already been well-demonstrated in animal and human studies.^37–40^

Our cohort reflects a stepwise approach in line with guidelines by El-Dib et al.^10^ with lumbar punctures systematically implemented as first-line measures in our center in 2020, escalation to tNSI when necessary, and shunt placement only after failure of temporizing interventions. Observational studies suggest benefit from early CSF drainage, while randomized trials—including ELVIS—remain inconclusive, though post-hoc analyses indicate potential magnetic resonance imaging and clinical advantages.^11,41–43^ In line with this evidence, and consistent with our previously published findings,^44^ we observed that cerebral oxygenation improved after tNSI in infants without prior lumbar punctures, underscoring the reversibility of PHVD-related hypoxemia through timely intervention.

Recent guidelines by El-Dib et al^10^ emphasize the importance of close ultrasonographic monitoring of ventricular size and timely intervention, while also monitoring clinical criteria without delaying action until clinical symptoms become apparent. The authors further highlight that NIRS could serve as an adjunctive tool for decision-making, considering the alterations in cerebral oxygenation described in previous studies.^10,13,14^ A recent study by our group assessed the effects of ventricular decompression on cerebral oxygenation 2 weeks before and after intervention, endorsing the early intervention with cerebrospinal fluid drainage via repeated lumbar punctures to reduce intracranial pressure and prevent cerebral saturations to drop below the threshold of 55% rScO_2_.^44^

In alignment with reported short-term alterations, our study highlights long-term changes in cerebral oxygenation, demonstrating significant differences between infants with IVH and those with IVH+PHVD, as well as among infants with an increasing PHVD burden. While the SafeBoosC III trial found no benefit of early NIRS-guided management in unselected preterm infants, our findings suggest value in targeted, prolonged monitoring in high-risk infants with IVH and PHVD.^45^

In addition to neurosurgical interventions, strategies to improve cerebral oxygenation should also be considered to mitigate the risk of secondary brain injury. For instance, increasing hemoglobin/hematocrit levels could enhance oxygen delivery and cerebral perfusion, particularly in infants with severe IVH or PHVD. Individualized oxygenation targets may be necessary for these high-risk infants. However, given the long-lasting alterations we observed, current rScO₂ thresholds— derived from early postnatal data—may require reevaluation.

### Strengths and limitations

This study benefits from a large cohort of preterm neonates with IVH (*n* = 154), spanning a wide range of IVH severity and PHVD burden, allowing a comprehensive evaluation. The prospective design further enhances the reliability of the data by minimizing bias. The extensive observation period until term-equivalent age represents one of the longest analyzed periods so far, providing valuable insights into long-term changes of cerebral oxygenation. Moreover, the large number of NIRS measurements strengthens the study’s robustness and the validity of its conclusions about IVH and PHVD trajectories.

Nonetheless, this study has several limitations: This single-center design limits generalizability, and the cohort is biased toward higher-grade IVH. Of 350 eligible infants with an initially even IVH grade distribution, only 154 were included, leading to uneven representation and selection bias. This reflects clinical practice, where limited device availability led to prioritization of infants with more severe IVH, reducing applicability to milder cases.

We also did not analyze differences between infants with different underlying IVH grades accompanied by PVHI, which may represent distinct pathophysiological subgroups with potentially different cerebral oxygenation patterns.

The relatively small subgroup of infants with PHVD not requiring intervention reduces statistical power and limits conclusions about the long-term effects of PHVD. Furthermore, a 10% minimum data availability per 1-h window was chosen as a pragmatic cutoff to balance data retention and quality, though this is suboptimal. The lack of detailed analysis of factors such as hemoglobin levels, medication use, and other clinical variables represents an additional limitation. An important methodological limitation is that the number of tNSIs was used as a surrogate for PHVD burden. Although clinically meaningful, this measure may not fully capture variability in disease severity and is influenced by institutional protocols and clinical decision-making. More standardized approaches, such as quantifying ventricular enlargement above defined thresholds or time spend above clinically relevant cut-offs, would provide a more objective assessment of PHVD severity and should be prioritized in future studies.

Finally, the lack of long-term neurodevelopmental outcome data limits the ability to fully assess the broader impact of IVH and PHVD. This was beyond the scope of the present study but will be addressed in subsequent analyses.

Future research should also focus on employing combined methods, such as NIRS, amplitude-integrated electroencephalogram, and magnetic resonance imaging, to enhance understanding of the mechanisms and effects of IVH and PHVD.

## Conclusion

Neonates with high-grade IVH exhibit a progressive decline in rScO_2_ during the first months of life, while those who develop PHVD show even greater reductions in rScO_2_ and increased cFTOE values. These findings reflect inadequate cerebral oxygenation, indicating persistent hypoxemia, increased oxygen demand, and an elevated risk of secondary brain injury.

In conclusion, our findings underscore the critical importance of monitoring cerebral oxygenation in preterm infants with IVH and PHVD. By demonstrating the impact of IVH severity and PHVD burden on cerebral oxygenation, this study offers valuable insights into the management of these high-risk neonates and identifies key areas for future research to improve clinical outcomes.

## Supplementary information

Supplementary Figure S1

Supplementary Table S1

## Data Availability

The datasets generated during and/or analyzed during the current study are available from the corresponding author on reasonable request.
