# Supplementary material for: Impact of posthemorrhagic ventricular dilatation on cerebral oxygenation in preterm infants with intraventricular hemorrhage
Source: Pediatr Res. 2026 Jan 8;99(4):1398–406. doi: 10.1038/s41390-025-04738-y (PMC13102702; doi:10.1038/s41390-025-04738-y)
Supplement: Supplementary file 1 — Supplementary Figure S1 [file 41390_2025_4738_MOESM1_ESM.pdf]

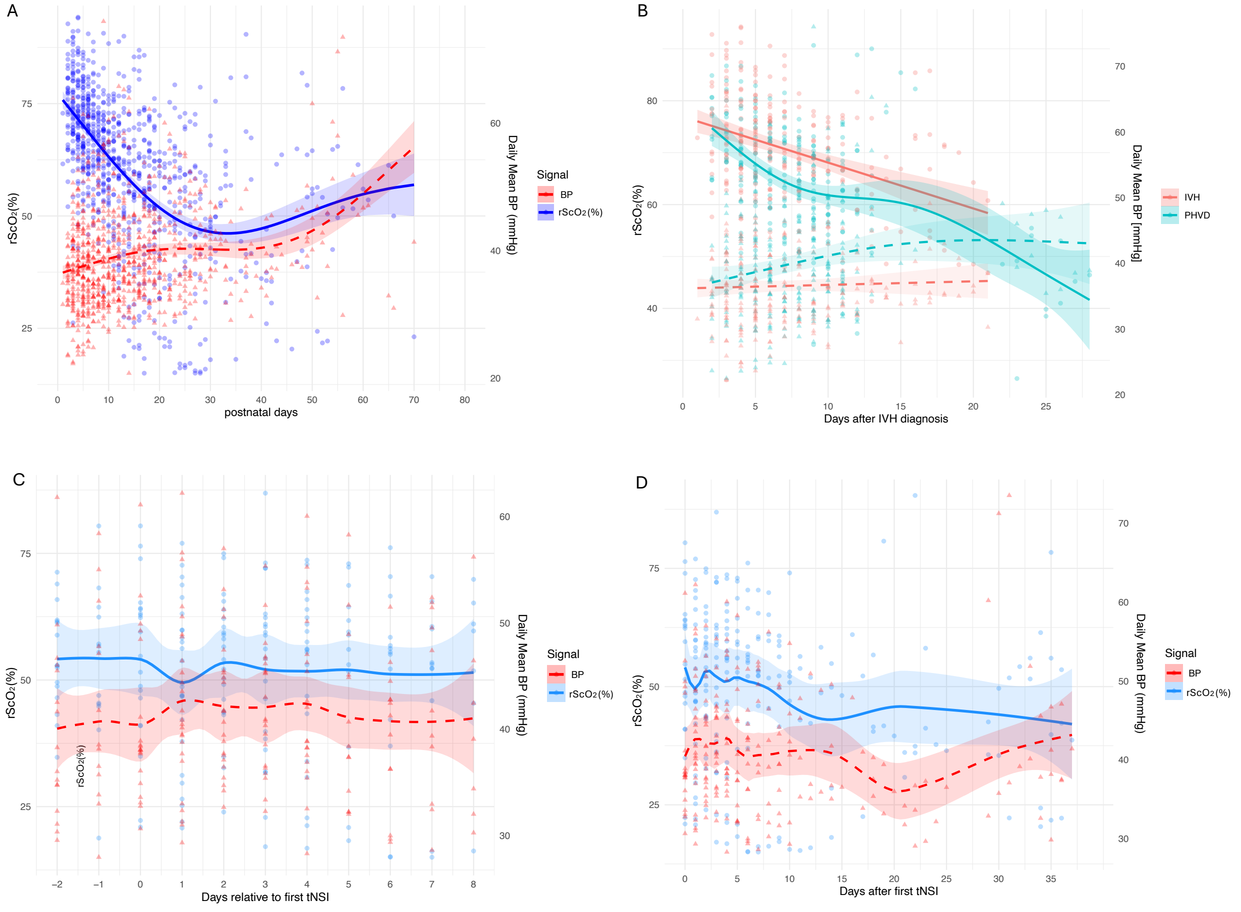

Supplementary Figure 1A-D. Association between mean arterial blood pressure (MAP) and cerebral oxygenation ($rScO_2$). (A) Across the entire study period (B) In the acute IVH phase (first 7 days after IVH diagnosis) (C) During the acute PHVD phase (−2 to +8 days around the first tNSI) and (D) in later follow-up (+39 days after the first tNSI). Solid/dashed lines indicate LOESS non-linear regression curves, with gray shading representing the 95% confidence interval.
