# Supplementary material for: Impact of posthemorrhagic ventricular dilatation on cerebral oxygenation in preterm infants with intraventricular hemorrhage
Source: Pediatr Res. 2026 Jan 8;99(4):1398–406. doi: 10.1038/s41390-025-04738-y (PMC13102702; doi:10.1038/s41390-025-04738-y)
Supplement: Supplementary file 2 — Supplementary Table S1 [file 41390_2025_4738_MOESM2_ESM.pdf]

Supplementary Table 1: Median $rScO_2$ values by IVH grade and postnatal age (in days)

Postnatal days	Grade I (n=12)	Grade II (n=23)	Grade III (n=29)	PVHI (n=25)	*p*
1-7	74.53 (64.31-81.22)	78.34 (74.07-82.99)	70.51 (67.56-77.89)	74.45 (68.01-78.23)	0.133
8-14	62.8 (60.50-73.36)	67.87 (62.88-73.76)	63.79 (56.40-73.00)	64.85 (52.45-71.88)	0.111
15-21	69.47 (63.96-77.32)	66.80 (60.24-73.82)	55.03 (52.33-63.44)	52.40 (34.68-56.64)	**<0.001**
22-28	79.91 (79.25-80.56)	63.74 (55.50-71.85)	59.34 (47.44-67.36)	52.95 (45.91-64.81)	**0.002**
29-35	60.71 (55.72-69.89)	60.88 (48.36-70.65)	57.99 (51.56-69.70)	49.32 (38.02-60.10)	**0.011**
36-42	61.80 (52.22-70.26)	68.08 (52.55-80.97)	63.51 (56.34-64.21)	48.19 (30.33-65.36)	**0.004**
43-49	72.83 (65.0-80.28)	55.67 (46.55-69.18)	66.56 (60.61-76.00)	61.97 (40.92-64.04)	**0.008**
50-63	66.07 (65.14-73.40)	65.35 (55.71-69.08)	64.28 (54.56-75.27)	59.07 (38.07– 65.73)	**0.047**
64-77	68.68 (64.45-74.39)	62.06 (59.80-79.82)	66.03 (55.33-77.56)	65.24 (46.26-70.33)	0.075
78-91	72.3 (66.73-82.20)	66.31 (55.67-71.76)	63.50 (49.25-68.86)	64.53 (39.02-71.13)	0.052
92-105	69.92 (55.79-75.18)	71.38 (66.39-73.57)	65.77 (44.35-71.11)	62.70 (41.51-68.91)	0.391
